# Increased HIV and other sexually transmitted infections in two health facilities in Northern Cameroon between 2021 and 2022

**DOI:** 10.4102/jphia.v16i1.690

**Published:** 2025-02-25

**Authors:** Patrice Djataou, Marceline D. Ngounoue, Georges Nguefack-Tsague, Jean de Dieu Anoubissi, Joséphine J.K. Kadji, Tiga A. Aline, Elise Elong, Moussa Djaouda, Alexis Ndjolo, Celine N. Nkenfou

**Affiliations:** 1 Laboratory of Systems Biology, Department of Biochemistry, Faculty of Science, ‘Chantal Biya’ International Reference Centre for Research on HIV/AIDS Prevention and Management (CBIRC), University of Yaoundé I, Yaoundé, Cameroon; 2 Department of Biochemistry, Faculty of Sciences, University of Yaoundé I, Yaoundé, Cameroon; 3 Department of Public Health, Faculty of Medicine and Biomedical Sciences University of Yaoundé I, Yaoundé, Cameroon; 4 National AIDS Control Committee, Yaoundé, Cameroon; 5 Laboratory of Systems Biology, ‘Chantal Biya’ International Reference Centre for Research on HIV/AIDS Prevention and Management (CBIRC), Yaoundé, Cameroon; 6 Department of Life and Earth Sciences, Higher Teachers’ Training College, University of Maroua, Maroua, Cameroon; 7 Department of Biological Sciences, Higher Teachers’ Training College, University of Yaoundé I, Yaoundé, Cameroon

**Keywords:** HIV, prevalence, new infection cases, hepatitis B, syphilis, insecurity, Northern Cameroon

## Abstract

**Background:**

Human immunodeficiency viruses (HIV) and acquired immunodeficiency syndrome (AIDS) remain a global public health problem. Other sexually transmitted infections (STIs) are aggravating factors.

**Aim:**

This study aimed to assess the prevalence and identify new cases of HIV and STIs, as well as their associated risk factors.

**Setting:**

Political insecurity in the northern regions of Cameroon has led to population displacement, weakening an already fragile health system.

**Methods:**

A cohort of 684 consenting participants from the north and far north were enrolled in 2021 and followed up in 2022. Socio-demographic variables and risk behaviours were collected. Anti-HIV Ab, hepatitis B surface antigen, *Treponema pallidum* haemagglutination tests were performed. The data were analysed using Epi Info 7.5.2. The associations between variables were evaluated using the Chi-square test with a 95% confidence interval.

**Results:**

The new cases of HIV rate and overall prevalence were 1.63% (95% confidence interval [CI]: 0.83% – 2.41%) and 3.8% (95% CI: 2.01% – 3.97%), respectively. New HIV cases increased from 0.27% (2017, Demographic and Health Survey [DHS]) to 1.63%. The prevalence of syphilis and hepatitis B was 1.03% (95% CI: 0.98% – 1.09%) and 4.56% (95% CI: 4.51% – 4.66%), respectively. Factors associated with HIV included religion (*p* = 0.027), unprotected sex (*p* = 0.006), sex with a sex worker (*p* = 0.00009), and co-infection with syphilis and hepatitis B (*p* = 0.033). New HIV infections may also be associated with population displacement.

**Conclusion:**

HIV infection, syphilis and hepatitis B are on the rise in the Northern Cameroon.

**Contribution:**

Future HIV prevention strategies should consider population displacement and HIV-associated infections such as hepatitis B and syphilis in order to secure achievements in HIV programme and further curb the burden of these infections in the country.

## Introduction

In 2020, there were still 1.3 million new human immunodeficiency virus (HIV) infections, despite considerable efforts to reverse the trend. The total number of people living with HIV worldwide was 37.7 million, with 20.6 million in Africa.^1^ There is currently no vaccine or curative treatment for HIV or acquired immunodeficiency syndrome (AIDS). Control of the pandemic still relies on prevention and surveillance. According to the Joint United Nations Programme on HIV/AIDS (UNAIDS) 95-95-95 strategic plan projected for 2025,^2^ 95% of people living with HIV should know their HIV status, 95% of all HIV-infected people tested should receive sustained antiretroviral therapy, and 95% of people receiving antiretroviral therapy should have sustained viral load suppression. Challenges remain in achieving all the three targets. Monitoring new infections is key to achieve the first target. This includes frequent and repeated HIV testing of negative individuals and identification of associated risk factors. Cameroon is one of the hardest-hit countries in sub-Saharan Africa with localised and generalised epidemics.^3^ Although overall HIV prevalence among adults continues to decline, from 5.4% in 2004^4^ to 4.3% in 2011,^5^ 3.4% in 2017^6^ and most recently 2.7% in 2018,^7^ continued surveillance is recommended. The data collected show that prevalence is not evenly distributed across regions. This is related to socio-cultural parameters such as religion, occupation, sexual behaviour, neighbourhood, traditions, standard of living, political security and the associated influx of refugees, or the strength of the health system.^8,9,10^ According to a World Bank report, 56% of poverty in Cameroon occurs in the northern regions alone, where Islam is one of the main religions. Human immunodeficiency virus prevalence is lowest in the north and far north regions of Cameroon (1.6% and 1.5%, respectively). In recent years, the terrorist group Boko Haram has established itself in the far north region of Cameroon. The aforementioned security problems have led to a significant influx of refugees and population displacement in the northern part of the country, which is threatening the health system in this zone. These disruptions could have an impact on HIV transmission in Northern Cameroon. We are investigating the reasons for this low prevalence in order to develop new HIV prevention strategies in conflict zones. Under these circumstances, this study aimed to assess the prevalence, and identify new cases of HIV and sexually transmitted infections (STIs), as well as their associated risk factors, in order to inform the development of more effective surveillance strategies for the epidemic.^11^

## Research methods and design

### Study design

We selected two of the most accessible hospitals in the north and far north regions of Cameroon as our research sites.

The study was conducted at the main entry points of the regional hospitals of Garoua and Maroua, located in the north and far north regions of Cameroon, respectively. The participants were tested at the outset of the study and again 1 year later, with a 12-month interval between tests (four HIV serologic windows [June 2021 – May 2022]). The minimum sample size was calculated using the following formula, which allows for a margin of error of 5%.^12^ Given the low prevalence of HIV in the northern regions of Cameroon, we employed the Epi Info 7.2 software to calculate the size of the population, taking into account the following criteria: the initial population size was considerable, with 50% of cases, an acceptable margin of error of 5%, a power of 80% and a confidence level of 95%. The data yielded 663 participants. Accounting for a loss to follow-up of approximately 3.2% or 21 additional participants, the total number of participants in the study was 684:


$$Z_{1-\alpha /2^{2}}\times P(1-P)/d^{2}$$ [Eqn 1]

*P* denotes the prevalence of HIV infection in north and far north, which is under 5%; Z_α/2_, value of the fractile of the normal distribution at 5% = 1.96; *d*, degree of precision = 2%; the calculated size is *N* = 663 participants.

Taking into account a loss to follow-up of 3.2% (21 participants), the minimum sample size was set at 684 participants.

The study included individuals aged 18–70 years of both sexes who had lived in the north and far north regions for at least 5 years and who came to the hospital for work, medical consultations, or as visitors.

### Sampling

#### Inclusion criteria

The study population comprised individuals aged between 18 years and 70 years, of both sexes, presenting at the hospital during the study period. Additionally, the study population included individuals who had resided in the north and far north regions for a minimum of 10 years.

#### Exclusion criteria

The exclusion criteria included the presence of any pathology (e.g., leukaemia) that would contraindicate a blood draw.

Recruitment was conducted in two phases. Phase 1 (Year 1) ran from 15 June 2021 to 13 August 2021, and Phase 2 (Year 2) ran from 30 May 2022 to 15 July 2022. During the first year of the study, 684 participants were enrolled and 5 mL of blood was drawn for the following tests: HIV1/2, hepatitis B surface antigen (HBsAg) and TPHA (*Treponema pallidum* haemagglutination assay). These STIs are known to exacerbate HIV infection or facilitate its transmission.

Socio-demographic data were obtained through the administration of a pre-tested questionnaire. Furthermore, the questionnaire encompassed data pertaining to population movements and the settlement of new individuals within the surrounding neighbourhoods.

For HIV1/2, the rapid diagnostic test was performed according to the national algorithm, using Determine Alere HIV1/2 Strips (Abbott Diagnostics Medical Co., Ltd. 357 Matsuhidai, Matsudo-shi Chiba, Japan) and confirmed using KHB (Shanghai Kehua Bio-engineering Co., Ltd.; www.skhb.com) tests according to the manufacturer’s instructions. Hepatitis B surface antigen test was performed using Fortress Diagnostic’s HBsAg rapid screening tests and confirmed using HBsAg CYPRESS diagnostics ELISA kits (Cypress Diagnostics: Langdrop, Belgium; www.diagnostics.be), according to the manufacturer’s instructions. Syphilis test (TPHA) was performed by SD BIOLINE Syphilis tests and confirmed by ELISA Bio-Rad (Bio-Rad 3, Marnes-la-coquette-France), according to the manufacturer’s procedures.

In the second year of the study, 676 of the 684 participants who had been enrolled in the first year were retested. Six participants were unable to reach the study site and two others had died. Individuals who tested positive in the initial assessment were not subjected to a second examination. The Chi-squared test was employed to ascertain whether the observed prevalence rates were consistent with the known prevalence rates. Additionally, new cases of infection were compared with the national incidence, and socio-cultural and sexual risk factors were analysed. Values with a *p*-value less than 0.05 were considered statistically significant.

### Ethical considerations

Ethical clearance to conduct this study was obtained from the National Ethics Committee for Human Health in Cameroon (No. 2021/06/85/CE/CNERSHSP). All participants who were recruited consented to participate in the study and provided a written informed consent form.

## Results

### Socio-demographic characteristics of the study population

A total of 684 participants were enrolled. The mean age of participants was 31.4 ± 2.4 years, with a standard deviation of 12 ± 1.5 years. The age group most represented was that comprising individuals between the ages of 18 years and 35 years, which constituted 62.33% of the total number of participants. The female cohort constituted 47.37% (*n* = 324/684) of the study population, in comparison to 52.6% (*n* = 360/684) of the male cohort. The mean age at which the 684 participants engaged in sexual intercourse for the first time was 19.66 years, with a standard deviation of 3.7 years. The minimum age was 12 years and the maximum age was 32 years. A total of 15.6% of the participants disclosed a history of sexually transmitted infection, including cases of syphilis (*n* = 41), gonorrhoea (*n* = 37), and chlamydia (*n* = 12). For a comprehensive overview of the data, please refer to Table 1. A total of 547 participants (80% of the total number surveyed) indicated that population movement had occurred in their neighbourhood over the past 3 years.

**TABLE 1:** Socio-demographic and clinical characteristics of the study population.

Description	Men			Women		
	Mean	*n*	%	Mean	*n*	%
Variables	-	360	52.6	-	324	47.37
**Age (years)**	30.1 ± 2.1	-	-	32.03 ± 3.3	-	-
**Religion**						
Muslim	-	129	-	-	97	-
Christian	-	231	-	-	227	-
**Education level**						
University	-	71	-	-	52	-
Secondary	-	239	-	-	128	-
Primary	-	43	-	-	18	-
Illiterate or coranic study	-	7	-	-	126	-
**Marital status**						
Married	-	117	-	-	142	-
Single	-	156	-	-	168	-
Divorced/widowed	-	87	-	-	14	-

### Prevalence and new cases of HIV, hepatitis B and syphilis in the study population

The prevalence of HIV, syphilis, and hepatitis B infections in the initial phase of the study was 1.90%, 4.09%, and 0.146%, respectively.

The incidence of new cases of HIV, hepatitis B, and syphilis infections was found to be 1.62% (95% confidence interval [CI]: 0.83–2.41), 4.72% (95% CI: 4.51–4.660), and 1.03% (95% CI: 0.98–1.09), respectively (Table 2). In comparison with the incidence of HIV in Cameroon, there has been a notable increase in new cases of infection (*p* = 0.007).

**TABLE 2:** Prevalence and new cases of HIV, hepatitis B, and syphilis in the study population.

Variables	HIV (%)	95% CI	Hepatitis B (%)	95% CI	Syphilis (%)	95% CI
Prevalence	3.5	2.01–3.97	6.28	5.88–6.67	1.16	1.15–1.18
New cases	1.62	0.83–2.41	4.72	4.51–4.66	1.03	0.98–1.09

HIV, human immunodeficiency virus; CI, confidence interval.

The global incidence of HIV-hepatitis B and HIV-syphilis co-infection rates was 0.29% (95% CI: 0.18–0.41), and 0.15% (95% CI: 0.08–0.24), respectively. In the initial phase of the study, there were no cases of HIV-syphilis or HIV-hepatitis co-infection.

New cases of HIV infection detected were referred to the treatment centre in the region as per test and treatment strategies. Cases of new syphilis infections were immediately managed by the medical staff. Hepatitis B cases were referred to the ‘Centre Pasteur Annexe de Garoua’ for follow-up.

### Risk factors associated with HIV infection

As shown in Table 3, the socio-behavioural factors associated with HIV acquisition were religion (Muslim, *p* = 0.027), unprotected sexual intercourse with a new partner (*p* = 0.006), and sexual relationships with sex workers (*p* = 0.00009).

**TABLE 3:** Risk factors associated with HIV infection.

Variables	HIV-		HIV+		*p*	Relative risk (95% CI)
	*n*	%	*n*	%		
**Number of sexual partners**	-	-	-	-	0.1000	1.040
None	36	100.00	00	00.00	-	-
1 partner	469	96.13	06	3.87	-	-
2 partners	114	97.39	03	2.61	-	-
≥ 3 partners	46	95.83	02	4.17	-	-
**Knowledge of the HIV status of the partner(s)**	-	-	-	-	0.3900	1.070
Yes	351	71.64	08	28.36	-	-
No	296	68.49	03	31.51	-	-
No partner	18	100.00	00	-	-	-
**Religion**	-	-	-	-	**0.0270**	2.360
Muslim	226	96.58	08	3.42	-	-
Christian	437	99.32	03	0.68	-	-
Other	02	100.00	00	00.00	-	-
**Past STI**	-	-	-	-	0.7600	0.970
Yes	99	98.02	02	1.98	-	-
No	566	98.43	09	1.57	-	-
**Unprotected sex with a new partner**	-	-	-	-	**0.0060**	**0.0060**
Yes	101	95.28	05	4.72	-	-
No	564	98.95	06	1.05	-	-
**Sexual relationship with sex worker**	-	-	-	-	**0.0001**	3.880
Yes	95	91.35	09	8.65	-	-
No	256	98.08	05	1.92	-	-

Note: Bold values highlights a significant difference between religion and HIV status, indicating that Muslims are at a higher risk of HIV infection compared to Christians.

HIV, human immunodeficiency virus; STI, sexually transmitted infection; CI, confidence interval; -, negative; +, positive.

## Discussion

The study revealed an overall HIV and/or AIDS prevalence of 3.5% (2022), which was statistically distinct (*p* = 0.008) from the 1.55% documented by the Demographic and Health Survey (DHS) in 2018 in these two regions. In addition, the incidence rate of new cases was found to be significantly elevated (*p* = 0.007) in comparison to the 0.24% reported by the DHS in 2018 in Cameroon. This high prevalence rate may be attributed to the study setting as it was conducted in hospitals. The study found that new cases of HIV infection was 1.63%, which is significantly higher than the national incidence in Cameroon (0.24% according to DHS in 2018) with a *p*-value of less than 0.001. The statistical analysis revealed that factors such as religion, engagement in sex work, unprotected sex with a new partner, and the presence of STIs (syphilis and hepatitis B infections) were significantly associated with this high incidence. These findings are consistent with previous studies conducted among pregnant women or blood donors.^13,14,15,16^ Recent studies indicate a re-emergence of syphilis^17,18,19^ (1.02% new cases with an overall prevalence of 1.16%). Syphilis is known to promote the acquisition of HIV through the sexual lesions it causes.^18,19,20,21^

The deficiencies in the health system and prevailing insecurity in these regions have the potential to exacerbate pre-existing health issues. The recent influx of refugees has further compounded these challenges, leading to increased promiscuity, poverty, and unsanitary conditions. A number of studies have focused on the impact of armed conflict and displaced populations, demonstrating the adverse effects of political instability on the HIV and/or AIDS response.^22^ Although reviews indicate insufficient evidence to conclude that armed conflict is associated with an increase in prevalence,^23^ our study, conducted on a smaller population, shows an increase in prevalence in the specific context of Cameroon. In Cameroon, the ratio of health centres to the population is below the World Health Organization (WHO) recommended standard of one centre per 10 000 inhabitants, with only one centre per 20 000 inhabitants.^11^ This ratio declines further when considering the population of displaced neighbours, which has a negative impact on the quality of healthcare.

Ankouane et al. conducted a study on blood donors at the Yaoundé Central Hospital and found a prevalence of syphilis to be 0.2%.^15^ Newman and colleagues reported a worldwide prevalence of treponemal infection to be 0.5% – 0.6%.^24^ Recent research suggests that syphilis is re-emerging globally.^21,25,26,27,28^ Treponemal infection has the potential to act synergistically with HIV, increasing infectivity.^18,19,20,29,30^ The reappearance of syphilis in regions with lower HIV prevalence in Cameroon is a cause for concern, as it suggests the possibility of new HIV infections.

The prevalence and incidence of hepatitis B were 6.28% and 4.72%, respectively. These data illustrate a reciprocal correlation between the occurrence of hepatitis B infection and HIV infection, as evidenced by the prevalence and incidence studies conducted in co-infected individuals.^31^ The dissemination of the disease can be attributed to socio-cultural and lifestyle factors, including the practice of eating in a common dish and the utilisation of shared needles for scarification. Furthermore, there is a notable reluctance to seek treatment, which has led to an increase in new infection cases caused by the hepatitis B virus.^32^ In order to prevent the spread of hepatitis B, it is essential that community education and counselling programmes be implemented. The age group with the highest infection rate was between 25 years and 35 years of age, which corresponds to the most sexually active age group, as evidenced by other national and international data.^2,33,34^ A higher infection rate was observed among Muslims (*n* = 8; *p* = 0.027) compared to Christians. These results align with those reported in a study conducted by Mboppi et al. in 2014 in Meyomessala, South Cameroon.^8^ The study demonstrated that the Islamic religion is more tolerant of polygamy and the establishment of a patriarchal society. In a recent epidemiological and observational study conducted in Zambia, Nesamoney and colleagues identified religion^35^ and ethnicity/race,^36^ particularly associated practices, as significant factors in understanding the trend of HIV infection among Muslims. One of the socio-behavioural factors that facilitates HIV transmission is having had unprotected sex (*n* = 5; *p* = 0.0009). A review of previous incidence and prevalence studies^25,37,38,39^ have demonstrated that factors that exacerbate infection or the occurrence of infection are typically associated with an individual’s sexual behaviour within society. Those engaged in sex work represent a subset of the population that is persistently exposed and vulnerable to infection,^25,36,40^ as well as individuals with disabilities.^41^ It can be reasonably deduced that individuals who engage in sexual activity with this group are at risk of contracting HIV and other STIs. The study demonstrated that men who engaged in sexual activity with female sex workers (*n* = 9) were more likely to contract HIV than those who did not associate with these women (*n* = 5; *p* < 0.0001).

In a recent WHO release, key facts on HIV were presented. These facts indicate that HIV continues to be transmitted in every country in the world. Furthermore, some countries have reported an upward trend in new infections after a period of decline (https://www.who.int/news-room/fact-sheets/detail/hiv-aids 13 july 2023.)

It must be acknowledged that the limitations of this study are because of the relatively small size of the population under investigation. Nevertheless, the observed trend is a cause for concern.

The participants were recruited from regional hospitals in the north and far north, which may introduce bias and potentially inflate the infection rate, as found in the study.

Although the study period of 12 months is relatively short in comparison to other multi-year impact studies,^42,43,44^ the observed trend is nevertheless significant.

## Conclusion

This study indicates that HIV infection is on the rise in the north and far north regions of Cameroon, primarily because of the rising prevalence of syphilis and hepatitis B infections, in addition to religious beliefs and risky sexual behaviour. Moreover, the findings were obtained in a context of heightened insecurity and population displacement. This trend may be replicated in analogous contexts in other countries where political instability is a pervasive issue. It is therefore of the utmost importance to prioritise the control of the epidemic through the prevention of new infections in order to sustain the achievements made thus far.
